# What should be the timing of surgical treatment of humeral shaft fractures?

**DOI:** 10.1097/MD.0000000000019858

**Published:** 2020-04-24

**Authors:** Şeyhmus Yiğit

**Affiliations:** Arthroscopy, Trauma, Pediatric Orthopedics, Department of Orthopaedics and Traumatology, Private Sultan Hospital, Diyarbakir, Turkey.

**Keywords:** conservative treatment, fractures, humerus shaft, surgical treatment, timing

## Abstract

This study aims to evaluate the timing of surgery in surgically treated humeral shaft fractures, to investigate the effects of surgical time on fracture recovery and complications.

This was a retrospective and observational study, based on patient data who underwent surgical treatment of humeral shaft fractures from January 2012 to January 2019. There were 52 patients (19 were women and 33 men) with traumatic humeral shaft fractures who were treated consecutively at our hospital.

There was a statistically significant difference in time to start physical therapy, time between surgery and bone union, and time between bone fracture and bone union. The mean time to start physical therapy in group 1 was 6.5 weeks (range, 5–12 weeks), it was 10 weeks (range, 6–14 weeks) in group 2 (*P* < .001). The mean time between surgery and bone union in group 1 was 14.58 weeks (range, 12–20 weeks), it was 17.4 weeks (range, 8–30 weeks) in group 2 (*P*: .009). The mean time between bone fracture and bone union in group 1 was 113.2 days (range, 86–114 days), it was 179.2 days (range, 89–355 days) in group 2 (*P* < .001).

Classically the first treatment option for humerus shaft fractures is conservative if there is no absolute surgical indication. Surgical treatment may be the first option if patients want to return to early everyday life. Delayed surgery means delayed physical therapy and this means delayed recovery and return to everyday life. In today's technology world, it should be discussed that the initial treatment of uncomplicated humerus shaft fractures is a conservative treatment.

What is already know on this topic

- Classically the first treatment option for humerus shaft fractures is conservative if there is no absolute surgical indication.
- Delayed surgery means delayed physical therapy and this means delayed recovery and return to everyday life.

What this study adds

- In today's technology world, it should be discussed that the initial treatment of uncomplicated humerus shaft fractures is a conservative treatment.
- As the time between humeral shaft fracture formation and surgical treatment becomes shorter, healing accelerates and patients do not remain separated from their long-term lives.

## 1 Introduction

Humerus diaphyseal fracture approximately accounts for about 3% to 5% of all fractures.^[1]^ The rate of union of humeral shaft fractures treated conservatively is 67% to 98%.^[2,3]^ Nonunion of the humerus shaft fractures will cause long-lasting pain, deterioration in quality of life, and loss of function requiring surgical treatment.

Traditionally, the first treatment of humerus shaft fractures was closed reduction and splinting. The indications of surgery are neurovascular damages, joint fracture elongation, polytrauma, open fractures, pathological fractures, and the failure of conservative treatment.^[4,5]^ Non-operative treatment is based on the ability to compensate the angular and rotational deformity of the shoulder. Sarmiento published a study on non-operative method using functional brace in 1977.^[6]^ Functional bracing is performed on an average of 11.5 weeks (range, 4–22 weeks).^[3,7]^ Long-term immobilization is required in non-operative method. This treatment method has several implications including delayed ROM loss in the joints and also delay in return to work. Furthermore, skin and soft tissue complications are observed out of 1% to 9.5% functional bracing.^[8,9]^ Recently, orthopedic surgical technology has recorded considerable progress which enables patients to be more active. The patients are no longer want to break away from their life for a long time.

This study aims to evaluate the timing of surgery in surgically treated humeral shaft fractures, to investigate the effects of surgical time on fracture recovery and complications. Our hypothesis is that, as the time between bone fracture and bone union increases, the risk of nonunion and other complications increases.

## 2 Materials and methods

This was a retrospective and observational study. It is based on patient data who underwent surgical treatment of humeral shaft fractures from January 2012 to January 2019. There were 25 patients in group 1 (patients undergoing initially surgery treatment) and 27 patients in group 2 (patients undergoing surgical treatment after conservative treatment). There were 52 patients (19 were women and 33 men) with traumatic humeral shaft fractures who were treated consecutively at our hospital. Their age was between 20 and 65 years. Patient data included age, sex, the mechanisms of injury, operation time, blood loss, bone graft used, duration of hospitalization, complications, starting time of the physical therapy, and duration of union. Patients with missing patient data, fractures with radial nerve palsy, multiple injuries, patients with pre-existing limb fractures, open fractures, and pathologic fractures were excluded from the study. Inclusion criteria was humeral shaft fractures without joint involvement.

All patients who had humeral shaft fracture were included in the study without taking attention to the fracture pattern (transverse, oblique/spiral) and fracture position (proximal, middle, or distal). Fracture patterns were classified according to O/OTA-system.^[10]^

To all patients who admitted to our hospital with fracture of humerus was informed that the first treatment was closed reduction and splinting in the humerus shaft fractures. The complications of surgical treatment and closed reduction treatment were explained. After conservative treatment, we told patients that fracture could be a loss of reduction or nonunion and this treatment could continue for a long time. There was no definitive indication for surgery in patients who underwent surgical treatment. Surgical treatment was applied first because all patients wanted to return to their daily life early. The patients in group 2 who received conservative treatment were treated with nonoperative treatment at the other center or in our hospital, because they did not want surgical treatment. During the follow-up period, the patients in group 2 was operated because of reduction loss, delay union, or nonunion

All patients were operated in supine position under general anesthesia. Anterolateral approach was used to access to fracture. When the elongation of the fracture was too distal, the radial nerve was isolated. Lag screws and a lag plate were used. The required length plates were used to position at least 3 screws proximally and 3 distally to the fracture. In cases where necessary, we received bone graft from the iliac crest. If there wasn’t enough otograft from the iliac crest, we used allogratf. All postoperative patients were immobilized with splint. We calculated the average blood loss with the Gross Formula.^[11]^ 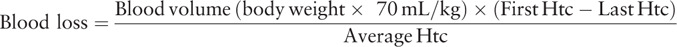


Clinical and radiographic follow-up was performed routinely in all patients at 2nd, 4th, 6th, 8th, and 12th weeks after surgery. Clinical and radiographic follow-up was continued if there was insufficient bone union. When adequate calluses occurred, the splints were removed and physical therapy was started.

The rate of complication for 2 groups was evaluated and compared on the basis of laboratory data. The patients were monitored for signs of infection, persistence of pain, improvement of elbow and shoulder ROM, neurovascular status, nonunion, and other complications.

The study was approved by the Ethics Review Committee of Dicle University Faculty of Medicine, Diyarbakir, Turkey. From all patients before surgery, we take surgical consent and permission to use data related to them. Written informed consent was obtained from all participants. All data were obtained without a personal identification document and made in accordance with the Declaration of Helsinki regulation.

SPSS24.0 statistical software was used to analyze the measured data. Mann–Whitney *U* test and Fisher exact test used to compare categorical data. Data results was carried out by the paired *t* test and *P* < .05, which was considered to be statistically significant.

## 3 Results

The mean follow-up period was 46.3 months (12–84 months). A total of 52 patients who underwent surgery for humeral shaft fracture met the inclusion criteria and were included in the study cohort. Thirty three out of 52 patients (63.4%) were men and 19 (36.6%) were women (*P*: .584). The mean age of group 1 was 39.6 years (range: 20–65 years), and the mean age of group 2 was 42.03 (range: 22–63 years) (*P*: .564) (Table 1).

**Table 1:**
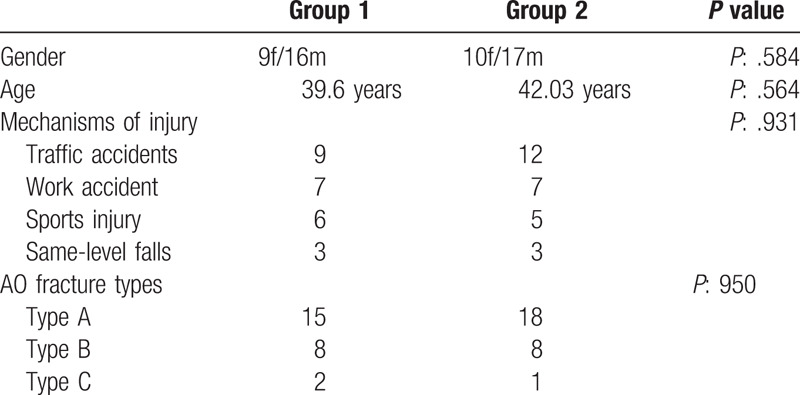
Patient data.

The mechanisms of injury were 31 (59.6%) traffic accidents, 14 (26.9%) work accident, 11 (21.1%) sports injury, and 6 (11.5%) same-level falls (*P*: .931). Group 1 had 15 (60%) Type A, 8 (32%) Type B, and 2 (8%) Type C fractures, group 2 had 18 (66.6%) Type A, 8 (29.6%) Type B, and 1 (3.7%) Type C fractures. There was no significant difference between the groups in injury mechanisms and AO fracture types.

There was a statistically significant difference in operative time, time in between bone fracture and surgical treatment and lastly blood loss. The mean time between bone fracture and surgical treatment (BF&ST) in group 1 was 5.9 days (range, 1–21 days), in group 2 was 57.4 days (range, 30–145 days) (*P* < .001). The mean operative time in group 1 was 92.2 minutes (range, 60–140 minutes), in group 2 was 119.2 minutes (range, 80–150 minutes) (*P* < .001). The mean blood loss was 281.2 mL (range, 200–400 mL) in group 1. It was 377 mL (range, 260–500 mL) in group 2 (*P* < .001). There were no significant differences in between the groups in bone grafting and hospital stay. Five cases of (30 cm^3^) otograft in group 1 and in group 2 7 cases of otograft (30 cm^3^) and 5 cases of allograft (60 cm^3^) was used (*P*: .057). The hospital stay was on average 3.2 days (range, 2–5 days) in group 1 and in group 2 it was 3.9 days (range, 3–5 days) (*P*: .04) (Table 2).

**Table 2:**
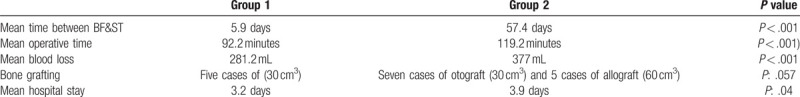
Operation data ∗ BF&ST (bone fracture and surgical treatment).

In both groups, there was a positive statistically significant difference in patients operated early between the time to start physical therapy, the time between surgery and bone union, and the time between bone fracture and bone union. The mean time to start physical therapy in group 1 was 6.5 weeks (range, 5–12 weeks), it was 10 weeks (range, 6–14 weeks) in group 2 (*P* < .001). The mean time between surgery and bone union (S&BU) in group 1 was 14.58 weeks (range, 12–20 weeks), it was 17.4 weeks (range, 8–30 weeks) in group 2 (*P*: .009). The mean time between bone fracture and bone union (BF&BU) in group 1 was 113.2 days (range, 86–114 days), it was 179.2 days (range, 89–355 days) in group 2 (*P* < .001) (Table 3).

**Table 3:**
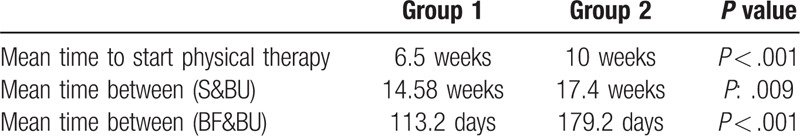
Healings data ∗ S&BU (surgery and bone union), BF&BU (bone fracture and bone union).

Group 1 had 3 cases of superficial infections, 2 cases of deep infections, 4 cases of radial paralysis, and 3 cases of delay union. Group 2 had 5 cases of superficial infections, 2 cases of deep infections, 8 cases of radial paralysis, and 2 cases of delay union. In Group 2, 2 cases of nonunion was observed (*P*: .719). Patients recovered by second surgery in other centers. Superficial and deep infections are healed with oral and iv antibiotic therapy. Because radial nerve exporation was done during the surgery, again the exporation was not done. After 3 months follow-up was performed with EMG. Radial nerve injuries improved in an average of 4.4 months (range, 3–7 months).

## 4 Discussion

The first treatment option for humerus shaft fractures is conservative if there is no absolute surgical indication. The indications for primary surgery of humerus saft fractures are floating shoulder, multiple trauma, neurovascular damage, nonunion, open fractures, and pathological fractures.^[12]^ If the non-surgical treatment requires prolonged immobilization and the patient does not want to wait so long or sometimes does not accept an acceptable residual deformity, conservative treatment cannot be performed. Surgical treatment may be the first option if patients want to return to early everyday life. The disadvantages of non-surgical treatment include joint stiffness due to long-term immobilization, general and particularly axillary hygiene problems, neck pain, pain and crepitation in the arm during the forearm movement, and difficulty of finding a comfortable sleeping position. Orthopedic surgeons can already perform surgical treatment to complicated humerus shaft fractures, such as open and fragmented fractures with nerve and vascular injury. In today's technology world, it should be discussed that the initial treatment of uncomplicated humerus shaft fractures is a conservative treatment. In our study, the shorter the time between the occurrence of humerus shaft fracture and the surgical treatment, the quicker we found the healing. The most important result of our study was that, there was a significant difference between the 2 groups in terms of time difference between humeral bone fracture formation and union of the humerus bone.

Factors such as smoking, excessive alcohol use, unnecessary anti-inflammatory drug use, infection, poor patient compliance, loss of integrity of soft tissues, insufficient immobilization, and inadequate fixation affect the healing of fractures. Although anatomical reduction is rarely achieved by nonoperative management of humeral shaft fractures, it is difficult to maintain reduction during shoulder and elbow movements.^[13]^ There is no consensus on the best treatment method in humerus shaft fractures in the literature. Retrospective studies by Mahabier et al^[14]^ found similar results between conservative and surgical treatments. It is also not clear that which one the best surgical technique is. Most of the patients (56.8%) underwent open reduction with internal fixation (ORIF) with plate fixation but intramedullary nails (IMN) and minimally invasive techniques were also involved.^[4]^ Recently Godinho et al^[15]^ have reported good radiographic and functional results using flexible nails such as Ender nails. The most important principle in fracture treatment is to provide rigid fixation that will allow early movement and facilitate bone healing. We applied open reduction and internal fixation to all patients with lag screws and a lag plate.

Arbeitsge-meinscheft for Osteosyntheses Fragen/Association for the Study of International Fixation (AO/ASIF) groups recommend broad steel plates of 4.5 mm in thickness for humeral diaphyseal fractures or nonunions.^[16]^ Ten or 11 hole steel plates are recommended for inserting ≥5 screws in the proximal or distal parts of osteoporotic nonunion fractures. Locking compression plates can be fixed in osteoporosis bone by 3 locked screws proximally or distally in osteoporotic humerus shaft fracture. However, it requires a long skin incision and extensive soft tissue dissection, which can prevent fracture healing.^[17]^ Hypertrophic nonunions can be healed with stable fixation. However, atrophic nonunions require both stable fixation and increasing biological response. Complex anatomy, unused osteopenia causes difficulty in the treatment of nonunion humerus shaft. When pseudoarthrosis surgery is planned, we should consider not only implant selection, but also the quality of bone. In group 2, there were more blood loss and prolongation of the operation. These results confirm our hypothesis. Delayed surgery means delayed physical therapy and this means delayed recovery and return to everyday life.

With regards to the trauma mechanisms, traffic accidents are in the first row similar to those found by Tsai et al.^[18]^ Tsai et al^[18]^ observed trauma mechanisms in the forms of traffic accidents (63.2%), same-level falls (15.1%), firearm injuries (11.8%), and falls from a height (11.3%). However, in our study we did not take open fractures due to firearm injuries.

The incidence of radial nerve palsy in humeral fractures is 11%.^[19]^ Approximately 3/4 of the nerve injuries occur in the primary (during the accident), and the rest (secondary to fracture manipulation). In our study, radial nerve paralysis and infection were seen as 17.3%. The incidence of infection in humeral fractures is 3%.^[20]^ In our study, radial nerve paralysis and infection were seen in 17.3% as more than in the literature. Although there was a high rate of radial nerve palsy according to the literature, all patients had improvement in follow-up periods. We suggest that radial nerve palsy will improve if radial nerve dissection is performed in humeral shaft fracture surgery.

There were several limitations in our study. This is a small group study which consists of 52 patients due to the problem of finding data in most of the retrospective studies. Patients with complete patient data were included in the study and the study was performed on the basis of this data. Pain and functional scoring of patients were not investigated because the study was based on examining the time between fracture formation and bone union.

## 5 Conclusion

Classically the first treatment option for humerus shaft fractures is conservative if there is no absolute surgical indication. Surgical treatment may be the first option if patients want to return to early everyday life. Delayed surgery means delayed physical therapy and this means delayed recovery and return to everyday life. In today's technology World, it should be discussed that the initial treatment of uncomplicated humerus shaft fractures is a conservative treatment.

## Author contributions

Şeyhmus Yiğit declare that he prepared the article together.

Şeyhmus Yiğit orcid: 0000-0002-7290-6798.

Şeyhmus Yiğit orcid: 0000-0002-7290-6798.
